# Grain Size-Related Strengthening and Softening of a Precompressed and Heat-Treated Mg–Zn–Ca Alloy

**DOI:** 10.3390/ma13020351

**Published:** 2020-01-12

**Authors:** Patrik Dobroň, Daria Drozdenko, Klaudia Horváth Fekete, Juraj Olejňák, Jan Bohlen

**Affiliations:** 1 Department of Physics of Materials, Faculty of Mathematics and Physics, Charles University, Ke Karlovu 5, 121 16 Prague 2, Czech Republic; 2 Nuclear Physics Institute, The Czech Academy of Sciences, 250 68 Řež, Czech Republic; 3 MagIC—Magnesium Innovation Centre, Helmholtz-Zentrum Geesthacht, Max-Planck Str. 1, D21502 Geesthacht, Germany

**Keywords:** Mg alloy, deformation-thermal treatment, yield asymmetry, Hall–Petch relationship

## Abstract

The impact of precompression, thermal treatment and its combination on the deformation behaviour of an extruded Mg–Zn–Ca (ZX10) alloy was studied with respect to a varied average grain size. The Hall–Petch plot was used to highlight the impact in a wide grain size interval. The initial texture of the wrought alloy was characterized by X-ray diffraction. Moreover, the evolution of microstructure and texture was provided by the electron backscatter diffraction (EBSD) technique. The obtained results indicate the strong contribution of deformation-thermal treatment on the resulting deformation behaviour. Particularly, after precompression and heat treatment, higher strengthening effect was observed in the reversed tensile loaded compared to compressed samples without any change in the Hall–Petch slope throughout the grain size interval. Unlike this strengthening effect, a reversed tension–compression yield asymmetry with higher strength values in compression has been obtained.

## 1. Introduction

The Mg–Zn–Ca alloys belong to the prospective metallic biomaterials due to their biocompatibility, biodegradability [1,2] and the Young’s modulus of the alloys being close to that of bones [3]. A low amount of alloying elements reduces production costs, and thus increases economic efficiency. In general, in wrought Mg alloys, beside a hexagonal close packed (hcp) lattice, deformation texture and the formation of precipitates contributes to the tension–compression yield asymmetry with higher strength values in tension than that in compression [4]. The grain refinement and/or texture weakening achieved by, for example, severe plastic deformation techniques or the twin-roll casting process also can lead to a lower yield asymmetry in Mg–Zn–Ca alloys [5,6,7,8].

Another concept for obtaining fine-grained microstructure and improving mechanical properties is using precompression and subsequent isothermal aging. It has been shown in [9] that application of this processing performed at 150 °C or 200 °C on the Mg–Zn–Ca (ZX10) alloy containing around 1 wt.% of Zn and 0.25 wt.% of Ca is beneficial for increasing the compressive yield strength (CYS). Furthermore, the level of precompression and the applied heat treatment strongly affects the tensile yield strength (TYS). Interestingly, after processing the alloy, the yield strength is higher in compression rather than in tension [10]. Similar mechanical properties are observed in natural bones, where CYS is found to be around 130–180 MPa, TYS varied between 80 and 150 MPa, and elongation to failure is 1%–7% [3]. Thus, the proposed processing seems to be useful for the production of the next generation of biomedical materials. This concept has been introduced for the ZX10 alloy with a homogeneous microstructure and an average grain size of 11 μm [9]. However, the grain size plays an important role in controlling the mechanical and corrosion properties of biomaterials [6,11]. Therefore, understanding the effect of precompression and applied heat treatment on the resulting mechanical properties with respect to grain size is essential.

Thus, the aim of the present study is to provide a comprehensive study on using precompression and subsequent isothermal aging in order to improve mechanical properties of the extruded ZX10 alloy with respect to the grain size. Particularly, the Hall–Petch plot is used to highlight the joined impact of precompression and precipitation on the mechanical properties of the alloy in a wide grain size interval.

## 2. Materials and Methods

The ZX10 (Mg + 1 wt.% Zn + 0.3 wt.% Ca) alloy was prepared by gravity casting and, prior to extrusion, the machined billets were heat-treated at 400 °C for 20 h in order to maintain a solid solution condition. The extrusion process for receiving a round bar with 10 mm diameter (extrusion ratio 1:25) was carried out either at 300 °C with an extrusion speed of 0.6 mm/s to produce a very fine-grained microstructure or at 400 °C with varied extrusion speeds of 0.5, 3.4 and 6.6 mm/s, respectively, to achieve microstructures with a range of different average grain sizes.

Deformation tests were performed using an Instron 5882 universal testing machine (Norwood, MA, USA) at room temperature and a constant crosshead speed of 10^−3^ s^−1^. All deformation samples were machined from the extruded bar with their loading direction along the extrusion direction (ED). Cylindrical samples for compressive loading had a diameter of 9.5 mm and a gauge length of 14 mm. For tensile loading and reversed tensile loading after precompression, samples with screw heads on both ends having a diameter of 5 mm and a gauge length of 13 mm were used.

Conditions for thermal and deformation-thermal treatment are based on our previous knowledge of the extruded ZX10 alloy (11 μm) [9,10]. In the present work, samples were precompressed up to 3% of plastic strain in order to introduce deformation twins inside the material. Further, using the same strain level of precompression provides a reference basis for a study of the deformation behaviour of the alloy with respect to grain size and the applied thermal or deformation-thermal treatment.

The heat treatment (HT) for 16 h at 150 °C followed by water quenching—isothermal aging (hereafter denoted as 16 h @ 150 °C)—has been adjusted to maximize the strengthening effect of precompressed material.

This HT has been applied to the as-extruded alloy in order to reveal its effect on the resulting deformation behaviour. Therefore, the following experiments have been performed:

- Compression of the as-extruded state
- Tension of the as-extruded state
- Compression of heat-treated (16 h @ 150 °C) samples
- Tension of heat-treated (16 h @ 150 °C) samples
- Furthermore, samples have been precompressed up to 3% of plastic strain and subjected to
- Compression
- Tension
- A heat treatment of 16 h @ 150 °C followed by compression
- A heat treatment of 16 h @ 150 °C followed by tension

The proposed procedure allows revealing an influence of intermediate treatment: sole HT, pre-deformation, combination of precompression and HT, on resulting deformation behaviour.

The global characterization of the texture of the alloy in as-extruded condition has been performed by using X-ray diffraction (XRD). A Malvern Panalytical X-ray diffractometer X’Pert (Malvern, UK) setup using CuKα radiation was employed to measure six pole figures on polished samples in reflection geometry to a sample tilt of 70°. To characterize the orientation distribution an open-source toolbox, MTEX (version 4.3.2, TU Chemnitz, Chemnitz, Germany, 2016) [12], has been employed for the recalculation and systematization of inverse pole figures.

For the microstructure investigation, typical metallographic procedures including grinding with SiC paper and polishing with diamond pastes down to a 0.25 μm particle size has been used. Finally, the surface was polished by means of ion beam Leica EM RES102 system (Leica Mikrosysteme, Wetzlar, Germany). The electron backscatter diffraction (ESBD) measurements were performed on the longitudinal sections parallel to the ED using Zeiss Auriga Compact focused ion beam scanning electron microscope (FIB-SEM) (Jena, Germany) equipped with an energy dispersive detector (EDAX, Mahwah, NJ, USA). EBSD measurements were conducted at a working distance of 9 mm with 10 kV acceleration voltage. The measured area and step size were varied with respect to the grain size of the investigated samples to get statistically relevant data. The orientation imaging microscopy (OIM) data analysis software (EDAX, version 7.3, Mahwah, NJ, USA, 2015) has been used for collection and processing of the obtained data.

## 3. Results

**Figure 1:**
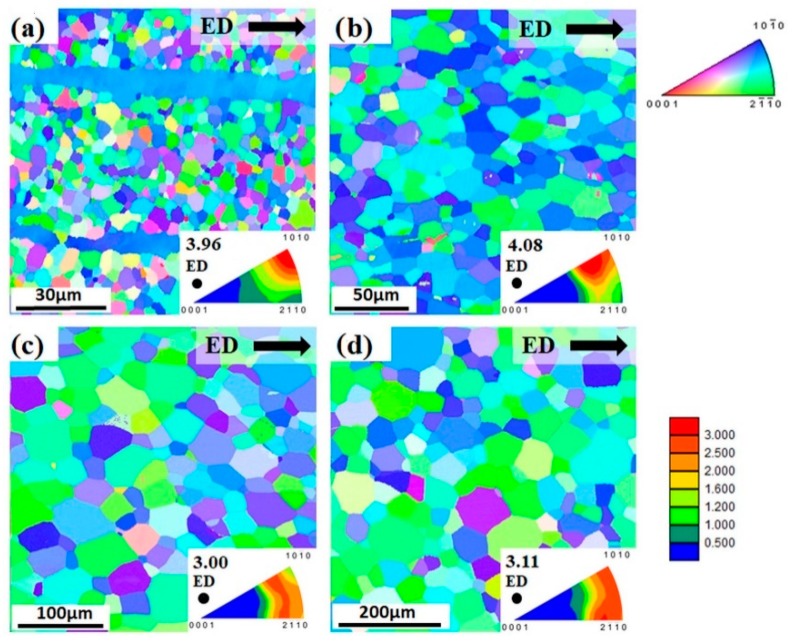
Initial microstructure of the extruded alloy: (**a**) ZX10-04, (**b**) ZX10-11, (**c**) ZX10-23, (**d**) ZX10-53 measured at longitudinal section (extrusion direction - ED is horizontal). Colour code used for orientation maps is represented along ED. Triangular inverse pole figure (IPF) in each subfigure represents texture of the alloy along ED (i.e., ED is perpendicular to IPF).

Figure 1 shows orientation maps from longitudinal sections of the extruded bars. Obviously, the average grain size varies as a result of the extrusion parameters. An increase of the extrusion temperature or the extrusion speed would typically result in enhanced grain growth during recrystallization, the latter as a result of increased deformation heating [13]. Thus, the average grain size of the investigated alloy varies between 4 µm and 53 µm with measurement error about 5%, as shown in Figure 1. In the case of the finest-grained extruded bar, the microstructure is not completely recrystallized and some large grains elongated along ED are still present in the alloy. In the three other conditions, fully recrystallized and homogeneous microstructures are found. To simplify a description of the different ZX10 alloy extrusions, the average grain sizes of the as-extruded state are added to the alloy label, i.e., a designation ZX10-04, ZX10-11, ZX10-23, and ZX10-53 are used throughout this work. The same designation is also used for the precompressed and/or isothermally aged samples in order to simply describe and follow the strengthening/softening of the individual alloy extrusions, i.e., deformation, thermal, and deformation-thermal effects on the grain size are omitted on the alloy label.

**Figure 2:**
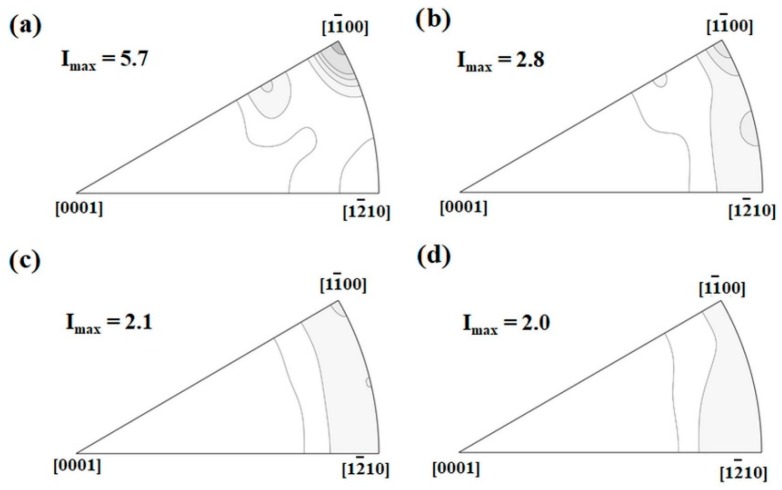
Inverse pole figures of the extruded alloy in initial as-extruded condition obtained by X-ray diffraction: (**a**) ZX10-04, (**b**) ZX10-11, (**c**) ZX10-23, (**d**) ZX10-53. The contour levels are 1, 1.5, 2, 3, 5, 7 m.r.d. (multiples of a random distribution).

The applied extrusion conditions result in textures with their basal planes mainly aligned parallel to ED, Figure 2. In the case of ZX10-04, a pronounced $\langle 10\bar{1}0\rangle$ texture component can be associated with the elongated grains, as shown in Figure 1a. In the other cases, there is no such strong alignment but a broader intensity distribution on the arc between the $\langle 10\bar{1}0\rangle$ and $\langle 11\bar{2}0\rangle$ poles. Therefore, the investigated extruded bars are characterized by comparable textures and they can be distinguished only by the grain size. It should be noted that results of texture measurements from XRD are in a good agreement with those recalculated from EBSD results, Figure 1 and Figure 2.

**Figure 3:**
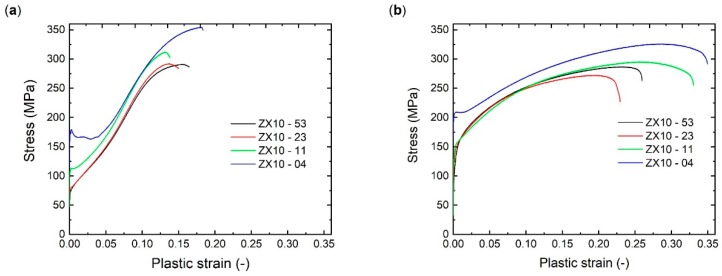
Compressive (**a**) and tensile (**b**) deformation behaviour of the extruded ZX10 alloy with respect to the average grain size.

Tensile and compressive deformation behaviour of the as-extruded state is presented in Figure 3. In the case of the ZX10-04 alloy, a pronounced yield plateau can be observed on both deformation curves, whereas the plateau is significantly longer on the compressive curve. Deformation curves of the ZX10-11, ZX10-23, and ZX10-53 alloy exhibit a similar trend throughout each loading direction. During tensile testing (Figure 3b), a continuous elastoplastic transition follows the increase in stress over a strain hardening range, with decreasing slope towards the maximum stress. Fracture strains appear grain size dependent, being higher at smaller grain size. In the case of compression tests (Figure 3a), after yielding, an increase of the slope during the strain hardening range is observed up to an inflection point before the slope decreases again. In earlier work this strain hardening behaviour has been associated with twin-dominated deformation rather than slip-dominated deformation operated in case of tensile testing [14]. If the heat treatment is applied before loading, there is no change in shape of the tensile or compressive curves compared to those observed in as-extruded state (Figure 3), and only a slight increase in the stress levels can be seen (deformation curves are not presented here).

**Figure 4:**
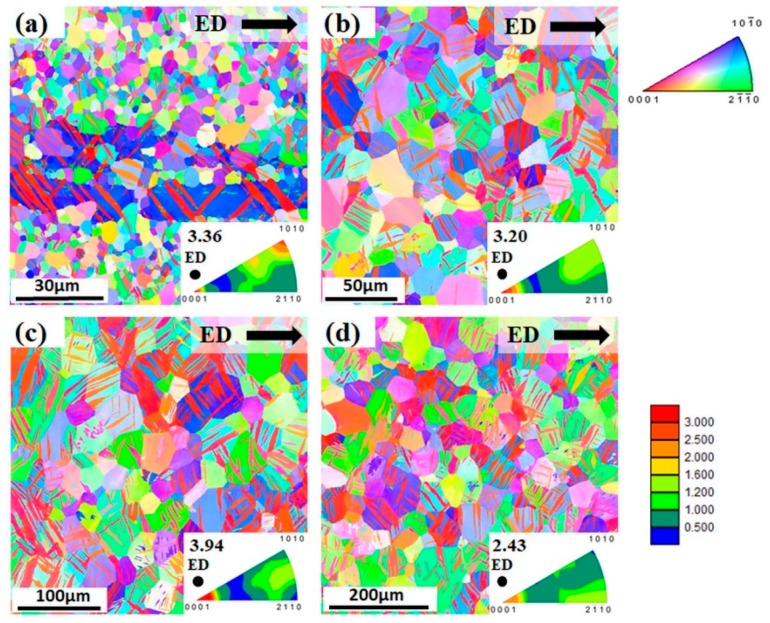
Microstructure of the precompressed extruded alloy: (**a**) ZX10-04, (**b**) ZX10-11, (**c**) ZX10-23, (**d**) ZX10-53 measured at longitudinal section (ED is horizontal). Colour code used for orientation maps is represented along ED. Triangular inverse pole figure (IPF) in each subfigure represents texture of the alloy along ED (i.e., ED is perpendicular to IPF).

To achieve 3% of precompression the following stress levels have to be reached for the particular extruded bar: 170 MPa (ZX10-04), 140 MPa (ZX10-11), and 124 MPa (ZX10-23 and ZX10-53). In other words, the stress level decreases with increasing grain size. During precompression, extension twins were formed in the microstructure (including sample with the finest grain size). The evidence of this type of twinning is presented in orientation maps in Figure 4 and it is also confirmed by the appearance of a new texture component with intensity at the 〈0001〉 pole compared to the as-extruded state, as shown in Figure 1.

**Figure 5:**
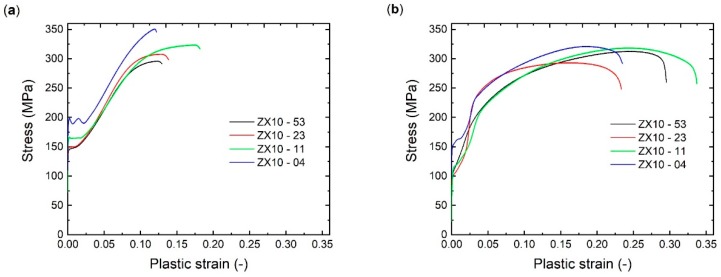
Compressive (**a**) and tensile (**b**) deformation behaviour of the extruded ZX10 alloy after pre- compression up to 3% of plastic strain and subsequent heat treatment (16 h @ 150 °C).

The stress–strain curves of precompressed samples with or without HT exhibit similar shape, only with an increase of stress levels with applying HT. Thus, deformation curves for samples subjected to precompression with subsequent HT (i.e., complete intermediate treatment) are presented in Figure 5. During recompression, all curves exhibit a yield plateau independently on the grain size and overall strain hardening behaviour for investigated samples is comparable, as shown in Figure 5a. Reversed tensile loading results in S-shaped curves for all investigated samples (Figure 5b) in contrast to the as-extruded samples, where rather concave shape is observed (Figure 1b). Moreover, this hardening behaviour at the beginning of the deformation (i.e., a significant S-shaped curve up to cca. 4% of strain) differs for the investigated samples.

**Figure 6:**
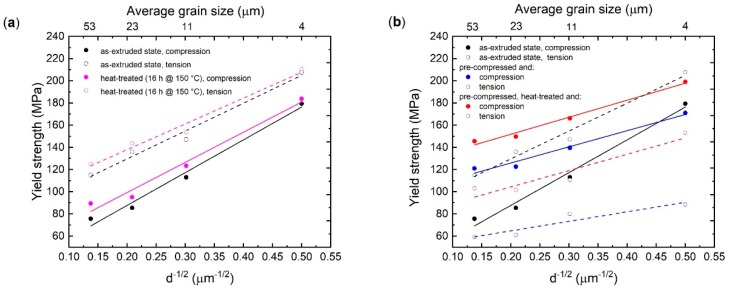
The Hall–Petch relation for the ZX10 alloy: (**a**) effect of HT for as-extruded condition; (**b**) effect of precompression with subsequent HT.

In order to capture the changes in the strengthening/softening behaviour caused by mechanical (predeformation), thermal or deformation-thermal treatment with respect to the grain size, a Hall-–Petch type interpretation can be used. Thus, the yield stresses are plotted vs. the inverse square roots of the average grain size in Figure 6. The yield strength asymmetry is revealed with higher stress values in tension rather than in compression (Figure 6a, Table 1). The grain-size related strengthening (i.e., Hall–Petch slope) is comparable for both loading directions (Figure 6a, compression—black solid line, tension—black dash line). The application of heat treatment only slightly reduces tensile–compressive yield asymmetry (Table 1) and changes the slope, caused by a larger increase in the stress in sample with higher grain size (ZX10-53).

In Figure 6b, the results for the precompressed samples are collected. For comparison, the results for the as-extruded conditions are repeated. The selected precompression level changes the slope for subsequently loaded samples. During recompression (blue solid circle), in case of the finest grain size (ZX10-04), the CYS is slightly decreased, whereas with the larger grained microstructures, the yield level is increased distinctly, which results in a lower slope (blue solid line) compared to that for compression of samples in as-extruded state (black solid line). It is noteworthy, that the compressive yield stresses of samples in precompressed state are only slightly lower compared to stresses applied to achieve 3% of strain during precompression.

For reversed tension of precompressed samples (blue open circle), a massive reduction of TYS compared to the initial state was observed. Particularly, the decrease is at least of 60 MPa in case of ZX10-53. Thus, a large reversed tensile–compression yield asymmetry was observed (Table 1). However, it should be noted that the slope for tension and compression of precompressed samples (Figure 6b, blue solid and dash lines) is similar.

The 16 h @ 150 °C heat treatment applied to the precompressed samples led to an increase in the yield strength during a subsequent compressive or tensile loading compared to that for sample after sole precompression (Figure 6b, red lines compared to blue lines, respectively). The increase was around 25 MPa for the compression and cca. 40 MPa for tension. Thus, the reversed tensile–compression yield asymmetry was reduced (Table 1).

## 4. Discussion

### 4.1. Effect of Extrussion Parameters

It is obvious, that even small changes in the process parameter settings during extrusion, i.e., varying the temperature between 300–400 °C and changing the extrusion rate between 0.6 mm/s to 6.6 mm/s (still rather slow extrusion) leads to significant changes in the resulting microstructures (homogeneity and a grain size range). In an attempt to keep the temperatures low that determine the grain structure development during recrystallization, the fine-grained microstructure still maintains an inhomogeneity due to an unfinished recrystallization process. Further grain growth can be enhanced by adding more thermal energy with further increase of the temperature or the extrusion rate [13,15]. The pronounced texture component observed at the $\langle 10\bar{1}0\rangle$ pole, in case of fined-grained material, is linked to the unrecrystallized elongated grains. Similar results have been observed in rapid solidified Mg alloy ribbons consolidated by extrusion, having a bimodal microstructure with an average grain size lower than 1 µm [16]. It was explained in [17], that this texture component is developed due to promoted basal and prismatic slip during the extrusion process.

Basically, all extruded bars (including the fine-grained one) exhibit a texture with preferential basal planes parallel to ED, typical for extruded Mg alloys, which favours deformation twinning when compression is applied along ED [18]. Thus, this type of texture contributes to a relatively strong tensile–compression yield asymmetry with higher TYS values, as shown in Figure 6a. The small deviation in the yield asymmetry for each tested sample regarding the average grain size can be associated with the orientation distribution variation in the initial texture.

### 4.2. Effect of Heat Tratment

There is a small effect of the heat treatment on the strengthening for the as-extruded samples, as shown in Figure 6a. It was documented in [9] that a heat treatment at 150 °C of this alloy promotes the formation of Mg_2_Ca precipitates in a form of short and thin plates aligned along the basal planes. These precipitates are distributed inside grains as well as along grain boundaries. The strengthening effect of basal Mg_2_Ca precipitates is reported to be very limited [19], which corresponds with the observed effect in present work, as shown in Figure 6a. It should be highlighted that in samples with a larger grain size, the precipitation strengthening effect is slightly higher compared to the finer-grained samples.

### 4.3. Effect of Precompression

Before evaluating the possible benefits of deformation-thermal processing, the effect of precompression on the following compressive or tensile properties is addressed. It is worth repeating that during precompression up to 3% of plastic strain, extension twins were formed in the microstructure (including sample with the finest grain size of 4 µm). The subsequent compression (Figure 6b, blue solid line) led to a slight reduction of CYS compared to precompression stress levels without any significant change in elongation to failure. This behaviour can be explained by an anelastic behaviour, which is commonly observed in Mg alloys during loading–unloading cycles [20,21,22]. The tensile deformation behaviour of the precompressed samples is strongly determined by the detwinning process. The shrinkage of the existing extension twins that formed during precompression is responsible for both: the significant decrease in the TYS (Figure 6b, blue dash line) compared to the initial state (black dash line), as well as the S-shape of the deformation curve, as shown as Figure 5b. It is not likely to associate this behaviour with a simple twin nucleation process, which determines the higher CYS of the as-extruded condition. In case of reversed tension, detwinning is related rather to the mobility of twin boundaries, which requires lower stresses compared to twin nucleation [23]. At the same time, twin boundary mobility also determines changes in hardening behaviour at the beginning of the tensile loading, i.e., significance of S-shape. A comprehensive study on reversed loading and resulting deformation behaviour in Mg alloys can be found e.g., in [24,25].

It is obvious that for the precompressed samples, the grain-size strengthening slope is determined by the precompression stress level. The precompression itself introduces mobile twin boundaries and dislocations. The mobile twin boundaries determine the deformation and do this similarity in both testing directions, tension and compression. Thus, a similar slope is observed (blue solid and dash line in Figure 6b), which is different to the as-extruded case (black solid and dash lines).

### 4.4. Effect of Precompression and Heat Treatment

In case of deformation-thermal-treated samples, HT leads to a significant increase in the yield stresses in both testing directions. The presence of dislocations acting as nucleation sites for fine particles to precipitate is likely to play a major role, especially if the effect is compared to the much lower increase of the yield stresses in case of the heat-treated samples without precompression in Figure 6a [26]. After precompression and HT, the yield stress increase is also more pronounced in tension (at least 40 MPa) compared to that in compression (cca. 25 MPa). However, HT does not introduce any specific grain size related (i.e., change in the slope) change for both tension and compression loading compared to samples after only precompression without HT. Thus, it is hypothesized that the yield determining mechanism—the onset for continuous twin boundary motion—is not changed due to HT in the investigated grain size range. This directly implies a distinct effect of dislocation precipitation additionally to a twin boundary pinning effect. During reversed tension, beside detwinning, dislocation slip remains the dominant mechanism, while during recompression, the twin nucleation is still the most favourable one. Thus, the higher YS shift in tension compared to compression indicates that precipitation at dislocations is main contributor to strengthening. The higher offset of tensile yielding leads to conclusion that the onset of detwinning could be also shifted to higher stresses as a result of precipitation. In case of the ongoing compression test, the nucleation of new twins can be assumed because from the textures point of view this still is the preferred mechanism to be activated at lower stresses [25,27]. The offset of additional 25 MPa is then related to the activation stress of twin nucleation in this specific condition and it is not grain size dependent, as shown in Figure 6b.

However, despite of the ability of this alloy to strengthening by HT, the reversed tensile–compression yield asymmetry persists throughout the investigated grain size interval (Figure 6b, Table 1). Thus, precompression in combination with a following HT allows for the adjustment of the tensile–compression yield asymmetry.

## 5. Conclusions

The extruded ZX10 alloy with an average grain size of 4, 11, 23, and 53 μm, respectively, has been prepared to study the influence of precompression and a subsequent isothermal aging on deformation behaviour, especially yield strength behaviour, as a function of the grain size. The following conclusions can be drawn from a comprehensive study of the individual processing steps:

- Heat treatment (HT) at 150 °C for 16 h leads to strengthening and consequently to a slight increase in stress compared to the as-extruded condition within observed grain size interval.
- However, using HT after precompression, the strengthening effect is significantly higher compared to that observed after HT of extruded bars.
- At the same time, HT reduces the impact of detwinning on yielding during reversed tensile loading.
- Precompression up to 3% of plastic strain slightly decreased the compressive yield strength and distinctly reduced the tensile yield strength in comparison with the initial state. However, the Hall–Petch plot is similar throughout the observed grain size interval for both loading conditions.
- The strengthening effect caused by heat treatment applied after precompression is more pronounced in tension than in compression without any change in the Hall–Petch slope throughout the observed grain size interval. Thus, there is no grain size dependence of strengthening.
- Deformation-thermal processing leads to reverse tensile–compression yield asymmetry with higher strength values in compression. By comparison of the yield strength in the initial state with that observed after deformation-thermal processing, it is evident that the increasing grain size has a positive effect on compressive yield strength and also reduces negative effect on tensile yield strength due to the twinning–detwinning process.

## Figures and Tables

**Table 1:** Tensile–compression yield asymmetry values in MPa for different states of the ZX10 extrusions. The yield asymmetry was calculated as the difference between the tensile and compressive yield strength value (standard deviations of stresses is 2 MPa), i.e., negative values represent the reversed tensile–compression yield asymmetry.

Designation	As-Extruded	Heat-Treated	Precompressed	Precompressed and Heat-Treated
ZX10-04	29	27	−83	−46
ZX10-11	34	31	−60	−55
ZX10–23	48	46	−62	−48
ZX10–53	39	36	−62	−43
